# Supplementary material for: B Cells and Double-Negative B Cells (CD27−IgD−) Are Related to Acute Pancreatitis Severity
Source: Diseases. 2024 Jan 5;12(1):18. doi: 10.3390/diseases12010018 (PMC10814478; doi:10.3390/diseases12010018)
Supplement: Supplementary file 1 [file diseases-12-00018-s001.zip › Supplementary Figure S1 - gating strategy.pptx]

## Slide 1
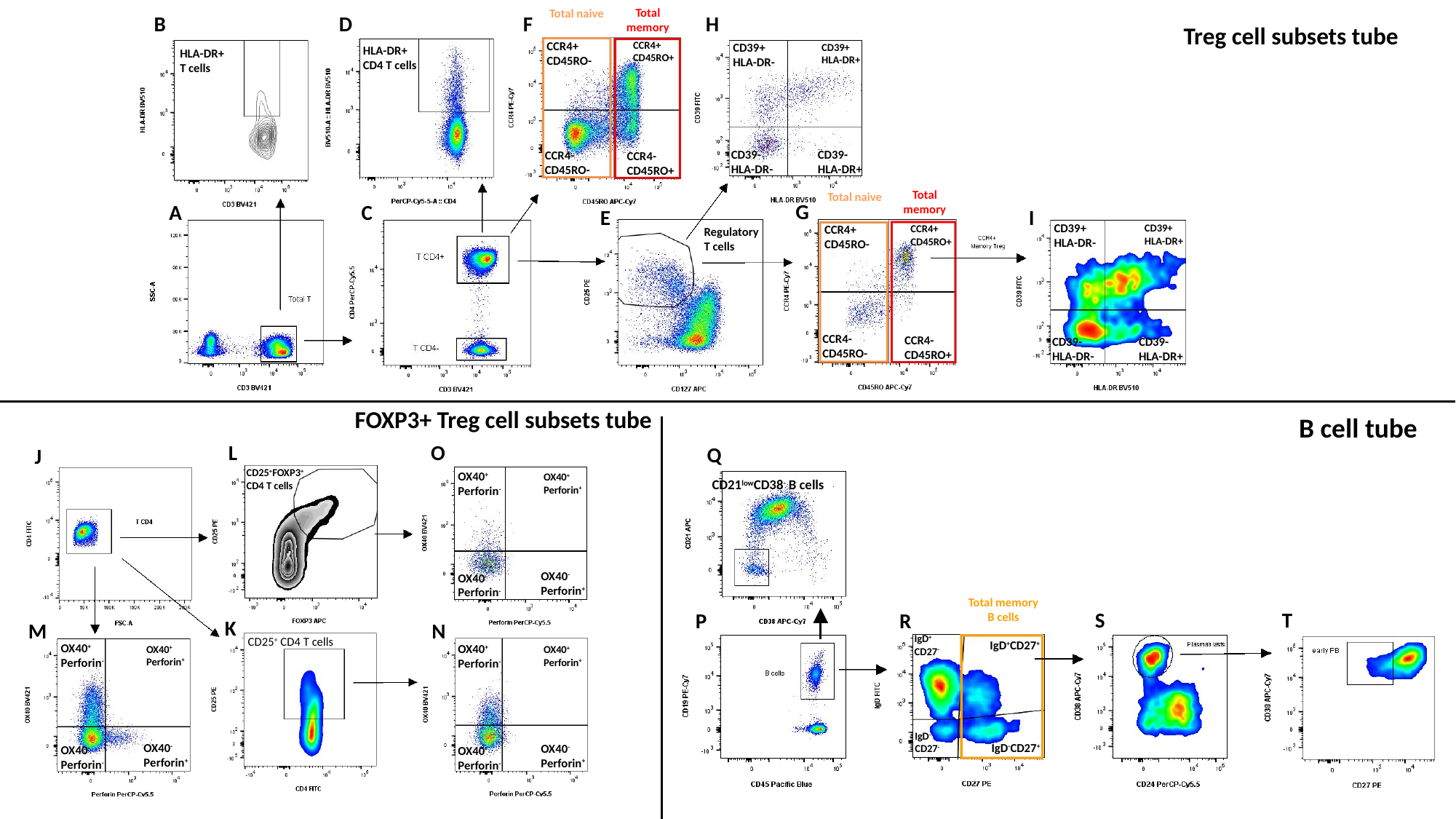

Total memory
Total naive
B
D
F
H
CCR4+
CD45RO-
CCR4+
CD45RO+
CD39+
HLA-DR-
CD39+
HLA-DR+
HLA-DR+ CD4 T cells
HLA-DR+ T cells
CD39-
HLA-DR-
CD39-
HLA-DR+
CCR4-
CD45RO-
CCR4-
CD45RO+
Total memory
Total naive
G
A
C
I
E
CD39+
HLA-DR-
CD39+
HLA-DR+
CCR4+
CD45RO-
CCR4+
CD45RO+
Regulatory T cells
CCR4-
CD45RO-
CCR4-
CD45RO+
CD39-
HLA-DR-
CD39-
HLA-DR+
Treg cell subsets tube
FOXP3+ Treg cell subsets tube
B cell tube
O
L
J
CD25+FOXP3+ CD4 T cells
OX40+
Perforin-
OX40+
Perforin+
OX40-
Perforin+
OX40-
Perforin-
K
M
N
CD25+ CD4 T cells
OX40+
Perforin-
OX40+
Perforin-
OX40+
Perforin+
OX40+
Perforin+
OX40-
Perforin+
OX40-
Perforin+
OX40-
Perforin-
OX40-
Perforin-
Q
CD21lowCD38- B cells
Total memory B cells
T
S
P
R
IgD+
CD27-
IgD+CD27+
IgD-
CD27-
IgD-CD27+
